# Expectant and New Mothers’ Use of Facebook Private Groups to Learn About Infant Sleep and Feeding Practices: Descriptive Qualitative Study

**DOI:** 10.2196/85640

**Published:** 2026-08-20

**Authors:** Rebecca F Carlin, Rachel Y Moon, Fern R Hauck, Margaret G Parker, Emma Forbes, Katherine Boguszewski, Ann Kellams, Eve R Colson

**Affiliations:** 1 Department of Pediatrics, Columbia University Vagelos College of Physicians and Surgeons, 622 West 168th Street, New York, NY, 10032, United States, 1 212-305-0345; 2 Department of Pediatrics, University of Virginia School of Medicine, Charlottesville, VA, United States; 3 Department of Family Medicine, University of Virginia School of Medicine, Charlottesville, VA, United States; 4 Department of Pediatrics, UMass Chan Medical School, Worcester, MA, United States; 5 Department of Obstetrics and Gynecology, Boston Medical Center, Boston, MA, United States; 6 Department of Pediatrics, WashU Medicine, St. Louis, MO, United States

**Keywords:** social media, parenting, safe sleep, breastfeeding, qualitative, health behaviors

## Abstract

**Background:**

Sudden and unexpected infant death is a leading cause of postneonatal death in the United States. Following recommended infant care practices such as safe sleep and breastfeeding reduces the risk of sudden and unexpected infant death. Parents use social media to help them make decisions about infant care practices.

**Objective:**

The objective of our qualitative study was to explore mothers’ perspectives on the acceptability of Facebook private groups for providing education on infant care practices.

**Methods:**

We conducted a descriptive qualitative study that recruited a purposeful sample of pregnant persons and mothers who recently gave birth; all were clients of the Special Supplemental Nutrition Program for Women, Infants, and Children in 1 of 2 states. We conducted in-depth interviews that were analyzed inductively through a systematic, iterative process using an interview guide. We asked participants about their perspectives regarding the use of Facebook private groups for learning about infant care practices, including sleep practices and breastfeeding. Data were collected until data sufficiency was reached.

**Results:**

Of the 22 participants, 13 (59.1%) were Special Supplemental Nutrition Program for Women, Infants, and Children clients from Rhode Island, and 9 (40.9%) were from Virginia. While participants’ use of Facebook in general varied, all either used Facebook private groups to obtain infant care practice guidance about safe sleep and breastfeeding or liked the idea of using specifically Facebook private groups to disseminate this information to pregnant persons and new parents. Participants reported using Facebook private groups for three main purposes: (1) information seeking, (2) the experience of engaging with Facebook private groups, and (3) social connectedness.

**Conclusions:**

Facebook private groups were endorsed by the participants for providing mothers with information about infant care practices. Participants outlined their reasons for and experience with the use of Facebook private groups, which can inform effective use of the platform for educational interventions.

## Introduction

Sudden and unexpected infant death (SUID), which includes sudden infant death syndrome, accidental suffocation and strangulation in bed, and ill-defined deaths, is a leading cause of infant mortality between 1 month and 1 year of age, accounting for approximately 3700 annual deaths in the United States in 2022 [1]. Black infants are more than twice as likely to die of SUID when compared to non-Hispanic White infants, and poverty remains an important risk factor [2]. Safe sleep practices are the only known way to protect against SUID. These practices include placing infants to sleep supine on a separate, firm, flat surface with no loose blankets or objects and room-sharing without bed-sharing. Despite widespread public health efforts to educate families on safe sleep practices and breastfeeding—behaviors that are crucial in reducing these deaths—the SUID rate in the United States has remained largely unchanged for over 20 years and may even be increasing most recently [3].

Parental decisions regarding sleep practices and breastfeeding are not based on knowledge of recommended practices from health care providers or other public health sources alone. Instead, mothers base many of their decisions about infant sleep and breastfeeding on information and advice from their social networks and increasingly on advice “crowdsourced” from others on the internet and/or social media [4]. Previous studies have shown that mothers want to weigh options for themselves, often trusting experiential advice from “people like me” more than evidence-based recommendations [5]. Consequently, social media now plays an important role in parenting decisions [6,7]. A recent study using 2021 Pew Research Center data found that the most popular social media platforms used by parents in the United States were YouTube (88%) and Facebook (79%) [8]. Mothers were especially likely to use Facebook, and 83% of the mothers who reported using Facebook did so daily [8].

Facebook private groups are communities people join based on common interests. They require moderator approval to view and participate in conversations. There are thousands of Facebook private groups for new or expectant parents based on city or neighborhood, profession, shared interests, or due dates. Facebook private groups have the option of having a moderator who can approve and remove posts and turn comments on or off. The moderators’ degree of expertise and active moderation vary widely across groups. Given the nearly ubiquitous use of social media to guide parenting practices and the popularity of Facebook among mothers, we hypothesized that Facebook private groups could be leveraged to improve safe sleep and breastfeeding practices by creating large networks to promote safer norms for mothers with young infants. Thus, the objective of our qualitative study was to explore mothers’ perspectives on the acceptability of Facebook private groups for providing education on infant care practices to inform the development of an intervention using this approach.

## Methods

### Design

We conducted a descriptive qualitative study [9,10] using a purposeful sample of pregnant persons and mothers with young infants to understand their perspectives on using Facebook private groups for education focused on safe sleep practices and breastfeeding. Understanding our target participant experience with Facebook private groups was the vital first step to developing an effective intervention using Facebook private groups to increase safe sleep behaviors and breastfeeding rates.

### Ethical Considerations

Boston University served as the data coordinating center for this study, and their institutional review board deemed this study exempt (H-41548). The University of Virginia (2675), Columbia University Irving Medical Center (AAAT8775), and Washington University in St. Louis (202108141) participated in analysis of deidentified data, with the latter two also deeming the study exempt from review. Participants were provided with an information sheet, and all questions were answered by Boston University staff. Verbal consent was obtained prior to study enrollment. All participants received US $50 gift cards for taking part. Interviewees were assigned a participant number, and no personal identifying information was included in stored or analyzed transcriptions.

### Sample

Clients of the Special Supplemental Nutrition Program for Women, Infants, and Children (WIC) who were pregnant or who recently gave birth were invited to participate in the study. All participants were at 27 weeks or more of gestation or had an infant younger than 6 months of age at the time of eligibility screening as they would have recent experience with using social media for information about infant care practices. To be eligible, participants also needed to be English speaking and own or have daily access to a Wi-Fi– or data-capable device on which they accessed social media. Participants did not need to use social media regularly or use Facebook to take part. Potential participants were excluded if they did not live or expect to live with their infant after birth or if the infant had or was expected to have a diagnosis that would impact infant care practices in a manner not compatible with study goals, including rare contraindications to breastfeeding or supine sleeping, such as use of medications contraindicated in breastfeeding, maternal HIV, or known spina bifida. Given the sensitive nature of these conditions, potential participants were asked whether their health care providers had advised that there was a contraindication to either practice rather than providing an exhaustive list of possible conditions. These eligibility criteria matched the criteria we intended to use in the larger intervention study.

WIC is an income-based program in the United States that provides supplemental nutrition vouchers for those who are pregnant or have dependents aged 5 years and under. WIC clients were chosen because the focus of our educational intervention was families who experience higher rates of SUID (eg, families who identified as being in a racial or ethnic minority group and/or with low income [11]). To be WIC eligible, one’s gross income must be at or below 185% of the US Poverty Guidelines [12]; thus, WIC eligibility is an easily verifiable proxy for income that does not depend on self-report. Our goal was to develop an educational intervention for clients from WIC sites nationally to decrease SUID risk factors and increase protective factors, including breastfeeding, in this population. We chose qualitative methods to better understand the participant experience with Facebook private groups as this source of information was vital to aid in the development of the intervention.

QR codes with links to eligibility surveys were available in WIC offices via flyers, so they were available to all clients. Those who completed the survey and were eligible were contacted by study staff to complete the informed consent process and schedule interviews. All interested eligible participants who responded to scheduling calls took part.

### Interview Guide Development

The topics for the interview guide were developed based on literature reviews of studies using social media groups for medical interventions and studies looking at maternal decision-making on infant care practices. The team also looked at existing new parent Facebook private groups for common topics. The topic guide was piloted and refined as the interviews continued to ensure that intended topics were addressed (Multimedia Appendix 1).

### Data Collection

We conducted in-depth semistructured interviews through videoconference from July 21, 2022, to February 1, 2023. Interviews were conducted in English by 2 trained interviewers (KB and CR). We used an interview guide asking participants about their perspectives regarding the use of Facebook private groups for learning about infant care practices, including infant sleep practices and breastfeeding. We used prompts to learn about any experiences they had with Facebook private groups, their thoughts about using the platform, and the trustworthiness of the information they received in such groups. Interviews lasted 30 to 60 minutes and were audiotaped and transcribed verbatim. Basic demographic and health data were collected during the consent process. Interviews were conducted until sufficient data were collected for the purposes of this study [13].

### Data Analysis

We analyzed data through a systematic, iterative process of data collection and analysis to inductively create categories and then themes. Each transcript underwent multiple reviews by 2 investigators (ERC and RFC), who performed the early readings and initial and iterative coding followed by theme development and creation of a codebook. The other investigators were provided with the codebook and several transcripts to review as we developed iterations of the codebook based on ongoing conversations about the findings to reach consensus [14]. The Dedoose software (SocioCultural Research Consultants) supported the organization of the analytic process [15].

## Results

### Overview

We conducted 22 interviews with 13 (59.1%) WIC clients from Rhode Island and 9 (40.9%) from Virginia. The median age of participants was 28.5 (IQR 23-32) years; 90.9% (n=20) of the participants identified as female, 36.4% (n=8) identified as non-Hispanic White individuals, 27.3% (n=6) identified as non-Hispanic Black or African American individuals, and 31.8% (n=7) identified as Hispanic; and 22.7% (n=5) were pregnant with their first child at the time of the interview (Table 1). A total of 4.5% (n=1) of the participants identified as nonbinary. While participants’ use of Facebook as a social media platform varied widely, all either used Facebook private groups to obtain safe sleep and breastfeeding information or liked the idea of specifically using Facebook private groups to disseminate this information to pregnant persons and new parents. They described a range of moderation practices in groups in which they were members, including moderators with expertise in topic areas and community volunteers. Participants reported using Facebook private groups for three main purposes: (1) information seeking, (2) the experience of engaging with Facebook private groups, and (3) social connectedness (Table 2). Illustrative quotes from each theme and subtheme can be found in Table 2.

**Table 1.** Participant demographics (N=22).

Demographic	Participants
State of residence, n (%)	
Virginia	9 (40.9)
Rhode Island	13 (59.1)
Age (y), median (range)	28.5 (18.0-38.0)
Gender, n (%)	
Woman	20 (90.9)
Genderqueer	1 (4.5)
Nonbinary	1 (4.5)
Race and ethnicity, n (%)	
Non-Hispanic Black	6 (27.3)
Non-Hispanic White	8 (36.4)
Hispanic	7 (31.8)
Missing	1 (4.5)
Educational level, n (%)	
Lower than high school	2 (9.1)
High school graduate	4 (18.2)
Some college	9 (40.9)
College graduate	4 (18.2)
Postgraduate degree	3 (13.6)
Number of live births, n (%)	
None	5 (22.7)
1	7 (31.8)
≥2	10 (45.5)

**Table 2.** Representative quotes from participants illustrating major themes and subthemes.

Themes and subthemes	Illustrative quotes
Information seeking	
Information quality and trustworthiness	“I like to look at the comments [to decide if I trust something]. I like to see if anyone else has tried it already. That’s why I realize I have to be on active Facebook groups and not just robots because I would want it to be where I see like if 100 moms tell me this works, I’m willing to try it....But I need to see that someone else was willing to give it a chance.” [Quote 1; participant 17] “I think Facebook is great because my husband uses Facebook...I used it the whole time I was pregnant for the groups of course. Much more easier to communicate with the different types of women who’ve been through similar situation like I have, and the security was a lot more trustworthy compared to Instagram who had a lot more bots from what I’ve noticed.” [Quote 2; participant 17]
Accessibility of information	“When I need a question, boom. Nobody has lives anymore, but that’s not just pregnant people, that’s people in general. So you can get a pretty quick response.” [Quote 3; participant 12] “Which is why I like Facebook groups because then you can go through and see the old posts and be like okay this is what I need right now. I don’t want to go searching through the 15,000 pages of pamphlets that I have. Here’s the information I need to know without a random Googling and having to try to trust what’s there.” [Quote 4; participant 20] “Yes, where I did have to wait to be invited by one of the admins. It was very helpful because obviously there’s like 30 or 40 of them. So it was very quick, and they asked relevant questions to make sure it wasn’t just someone random trying to join in.” [Quote 5; participant 17] “It’s good because everybody has a Facebook it’s more like a nationwide thing as to where if you do need the information it’s easily found just by looking up certain groups.” [Quote 6; participant 37]
Specific topics sought	“I most got tips on how to prepare for breastfeeding and how to prepare myself when even breastfeeding does not work. It was a lot more better than truthfully what I got from my military hospital. So it gave me a bit more experience and I knew it was being done right because I’ve had a lot of people come and tell me this must not be your first child because you know how to properly hold the child because like most first time moms don’t. I got a lot of great tips from that.” [Quote 7; participant 17]
Experience of engaging with a Facebook group	
Feelings that come from participating	“I don’t know how to say it, but we get judged on the way we treat our babies because, especially like—and it’s mostly other women like us that had kids before. So we are judged a lot and we are always doing stuff and people sometimes don’t appreciate moms and we should be more appreciative.” [Quote 8; participant 3] “I feel like the closed groups have always been more polite, more well-mannered, you know, because there’s always those people out there who are—have got to be rude and condescending and you know look down on people and they don’t know the like everybody has their own situation, you know like there’s just always one of those people there who has to be a negative Nancy.” [Quote 9; participant 4] “I feel moderation is more important than the actual content of the posts. So long as everything is moderated and it’s not necessarily censored but it’s done in the right mindset, then it’s easier to get along with their group to appreciate what’s going into it rather than it just being meme central and turning very quickly into something you don’t want to engage in.” [Quote 10; participant 13]
Engagement with a Facebook group	“I know there’s a lot of people who don’t feel comfortable um disclosing information, like something that they would feel ashamed of, you know, being a first-time mom and I feel like if you do feel ashamed of something that you don’t know what’s going on and you need the advice, I feel like having an anonymous post is perfect because then you can get the advice or experiences that you need without having to feel like you’re being a bad mom or you know, being a bad parent in that sense.” [Quote 11; participant 6]
Social connectedness	
Expanding social network	“Well, one thing about going to Facebook for stuff like that is you’ll get opinions from different people, from people all over actually. So it’s not just geared to one type of population or something like that, but it would give opinions and thoughts of a variety of different people.” [Quote 12; participant 10]
Community building	“Yeah, I was definitely a lot lonelier when I had a newborn. I wasn’t working either. So that was my only adult time was these other moms on the internet who were probably in the exact same phase that I was, but right now I can get out of the house, my kids are older, they’re in school, they’re independent. So I have a little more flexibility and freedom, especially with working. I can go into the office and have adult time.” [Quote 13; participant 14] “Everyone’s going through the same thing at the same time. You’re always talking to people who are either new moms or pregnant, trying to learn or trying to better themselves for their kids, which is the kind of environment you want to foster.” [Quote 14; participant 13]

### Information Seeking

Three subthemes were developed from descriptions of using Facebook private groups for information seeking: (1) quality and trustworthiness of the information; (2) accessibility of the information; and (3) ability to find information about specific topics, such as product information, parenting tips, tricks or hacks, and parenting styles or approaches.

#### Quality and Trustworthiness of the Information

Many participants noted that the information they received on Facebook private groups felt more trustworthy because it came from real people with similar experiences and the shared goal of being better parents (quote 1 from participant 17 in Table 2). They generally found that the information was both practical and honest. Some referred to trusting advice on specific questions they asked and on products others had used. However, others were more skeptical of any advice from social media, including from Facebook private groups, because they thought that they were likely based on information from, for example, bots or people who could personally gain from the advice they give (quote 2 from participant 17 in Table 2).

#### Accessibility of the Information

Participants liked the accessibility of Facebook private groups and often noted that they provided a venue where they could have their questions answered quickly at any time of day (quote 3 from participant 12 in Table 2). They also liked the searchability of the groups so they could find advice without actively asking (quote 4 from participant 20 in Table 2).

Some found that Facebook private groups, though closed, were still easily accessible without a long wait and liked that the vetting process provided some assurance about who was in the group (quote 5 from participant 17 in Table 2). Several participants also mentioned that many people already know about Facebook, which could facilitate quick access (quote 6 from participant 37 in Table 2).

#### Specific Topics Sought

Facebook private groups were viewed by participants as an easy way to ask specific questions to receive targeted responses. Parenting hacks, product recommendations, breastfeeding advice, and parenting styles were frequently referenced by participants as sought-out information on Facebook private groups. Pregnant persons also wanted to know whether symptoms they were feeling were experienced by others (quote 7 from participant 17 in Table 2).

### The Experience of Engaging With Facebook Private Groups

The experience of participating in Facebook private groups played an important role in participants’ willingness to use Facebook for a group whose primary objective was to provide education to parents, even if they did not otherwise use the platform. The 2 subthemes that were developed were feelings experienced while participating and different ways of engaging with such groups.

#### Feelings That Come From Participating in Facebook Private Groups

Participants frequently referenced negative feelings, most notably judgment and embarrassment, which can come from social media in general (quote 8 from participant 3 in Table 2), and felt that Facebook private groups provided more privacy and positive experiences with support rather than judgment, more honesty, and better social manners than nonprivate Facebook groups or other social media venues (quote 9 from participant 4 in Table 2). Thus, participants were interested in and sometimes actively sought Facebook private groups where they would feel more comfortable. They credited group moderation, screening of members, and the shared experience of members for this more favorable experience (quote 10 from participant 13 in Table 2).

#### Engagement With Facebook Private Groups

Participants liked that Facebook private groups offered the ability to engage in multiple ways. Some participants liked posting, whereas others preferred to only comment on others’ posts or more passively read and “like” posts. Most felt that the ability to post anonymously created a safer space with more positive feelings (quote 11 from participant 6 in Table 2). Some participants also said that they preferred to engage with moderated groups so that the conversation remained civil.

### Social Connectedness

Participants overwhelmingly felt that taking part in groups fostered social connectedness through the subthemes of community building and expanding social networks at a time that can be isolating.

#### Expanding Social Networks

Those participants who had used new parent groups or other affinity groups before reported that they were able to expand their social network and connect with other new parents. Participants also noted that, while parents in these groups share that they are all in a similar phase of life, because the groups are online, they are often more diverse than their personal social networks and, thus, could provide different ideas and perspectives from those of their families and close friends (quote 12 from participant 10 in Table 2).

#### Community Building

Shared experience was often cited in building a dynamic community in Facebook private groups. Participants described Facebook private group communities as providing support, understanding, and advice. Participants sought this type of community more at different times in their lives, with some describing needing it more with their first child, whereas others needed it more during later pregnancies due to the loss of a supportive parent or family member or moving away from previous supports (quote 13 from participant 14 in Table 2). Several participants noted the opportunity to build community around shared experiences (quote 15 from participant 13 in Table 2).

## Discussion

### Principal Findings

For this study, we set out to understand the perspectives of pregnant persons and new mothers regarding the use of Facebook private groups for learning about infant care practices, with a focus on those that reduce the risk of SUID, namely, safe sleep practices and breastfeeding. We discovered that, regardless of their feelings about using Facebook in general, participants expressed openness to and excitement about using Facebook private groups to learn about infant care practices. Many participants described Facebook private groups as providing trustworthy and easily accessible information and a more positive engagement experience compared to other social media and assisting in expanding their social networks. Furthermore, many felt that Facebook private groups created opportunities for social connectedness and support during a difficult transition period. Participants saw particular value in the shared experience and connectedness of the private group and its ongoing conversation. Despite reservations about Facebook in general, they expressed trust in the advice of other parents in Facebook private groups that are monitored over unmoderated material across social media.

Our findings on the acceptability of Facebook private groups for a parental health intervention are consistent with those of existing literature. Previous qualitative studies have primarily looked at the experience participants had in Facebook private groups or analyzed the transcripts of existing Facebook private groups. Groups have been viewed positively and described as a source of support and advice for parents with children with rare or challenging diagnoses, for childhood obesity prevention [6,16,17], for infant health information [18], for breastfeeding support [19], and for parents during the COVID-19 pandemic [20]. The themes that were generated through our study regarding why participants wanted to use Facebook private groups are also consistent with those found in previous literature on why new parents use Facebook private groups and the internet more broadly [7].

Our results also help further elucidate the reasons why we believe Facebook private groups facilitated by health care professionals have the potential to have a powerful impact on safe sleep and breastfeeding practices. Many of our participants acknowledged the postpartum period as a vulnerable time where they felt isolated and looked for easily accessible support and advice. Our participants described the support they received from closed groups as helping establish trust in the advice they received. While we hypothesized that they would come to Facebook private groups specifically for this peer support, the quality of moderation of the group was also valued by many participants who wanted to ensure that the advice was reputable. This is consistent with a study looking at Facebook private groups for parents with children with rare disorders, which found that 79% of participants would have liked a physician or medical professional to be in the group [21]. Thus, physician advice provided in a support group is likely to be both accepted and highly trusted.

Strengths of this study include sharing the perspectives of pregnant persons and mothers who are WIC clients, representing a group that is at high risk of SUID. We included participants from 2 cities. Similarly to all qualitative studies, our findings are not generalizable, and further research should explore the perceptions of caregivers in other demographic and geographic groups.

### Conclusions

Although their experiences with and opinions on Facebook vary, pregnant persons and mothers of young infants use and generally feel positive about Facebook private groups. Thus, these groups can serve as a useful platform for an intervention to encourage safe sleep and breastfeeding by providing education, support, and social connectedness during the postpartum period.

## Supplementary material

10.2196/85640 Multimedia Appendix 1 Interview guide.

## Abbreviations

SUID: sudden and unexpected infant death
WIC: Special Supplemental Nutrition Program for Women, Infants, and Children
