# Supplementary material for: Expectant and New Mothers’ Use of Facebook Private Groups to Learn About Infant Sleep and Feeding Practices: Descriptive Qualitative Study
Source: JMIR Pediatr Parent. 2026 Aug 20;9:e85640. doi: 10.2196/85640 (PMC13492726; doi:10.2196/85640)
Supplement: Supplementary file 1 [file pediatrics-v9-e85640-s001.docx]

**Supplement 1: Client In-Depth Interviews Guide and Questions**

1. Introduction

A. Welcome and introduction of researchers/moderator.

Thanks for participating.

Agenda setting (time allotted, etc).

We will be recording this interview so that we can transcribe it for review later. We will delete any information that could directly identify you from the transcript.

Reimbursement $50 gift card – emailed within approximately 7 days.

B. Overview of the topic

You were selected to participate because you are pregnant or the mother of a young baby.

We hope to learn about your use of social media for creating, sharing, and/or getting information, advice, and support during your most recent pregnancy, about caring for your baby, and about being a parent.

We are asking these questions because we are going to be designing private Facebook groups (or other social media) for pregnant women and new mothers. This group will provide pregnant and new mothers information on infant care practices like safe sleep and breastfeeding recommendations. We are getting information from pregnant women and new mothers to help us design the information that will be shared with the group and how that information will be shared.

C. Guidelines for a successful interview

There are no right or wrong answers, *just different points of view*.

Is it okay if we are on a first-name basis?

2. Ice Breaker

Can you tell me a bit about yourself? Are you pregnant, or have you recently given birth? when are you expecting? If you have a baby, how old is your baby? Do you have other children and what are their ages?

3. Social Media Questions:

*Since you are a parent of a(n) infant(s)or soon will be, we are interested in your social media use. By “social media,” we mean sites that you visit using the web or applications (apps), such as Facebook, Instagram, Twitter, Reddit, Tik Tok, YouTube or others to create content, share thoughts or get information.*

*Specifically, we want to know about your use of social media for creating, sharing and/or getting information, advice and support during your most recent pregnancy, about caring for your child and about being a parent.*

*I am going to start talking just about Facebook. We are planning a study looking for the best way to provide information to parents about safe infant sleep and breastfeeding. We plan to use a Facebook group.*

**1)In general, what do you think about using Facebook groups for providing information to parents about safe infant sleep and breastfeeding?**

Prompts to consider

What do you like about using FB for this?

What do you not like about using FB for this?

What would you think about joining a closed group where only people invited to the study could join?

Tell me about any experience you have had with closed FB groups.

Tell me why you joined this group, how long you stayed, what you learned.

What makes you decide to trust a group? How do you decide that the information in the group is trustworthy?

What would make you comfortable posting in a group?

How do you feel about anonymous posting?

You say you do not use FB yourself

Tell me why you do not use FB.

Tell me what could get you to join FB.

**2)What would be a good way to recruit you or other WIC clients to join this group?**

Prompts to consider

If your WIC center has had special programs, how do parents get invited?

Tell me more about that?

What works and what does not work with getting that information.

Describe any examples you have where you joined a program.

QR codes (available at the WIC centers, on materials or elsewhere)

Receiving text messages or phone calls from WIC staff

Frequency of reminders to join/incentives (e.g. number of entries in a lottery)

Other ideas

**3)We have created and tested short videos about safe sleep and breastfeeding.**

Prompts to consider

How often do you think it is useful to share new videos? Why?

What is the best way to get the participants to watch the videos? Why?

Besides videos, what else should we share?

Infographics, interactive modules or other materials, handouts

What topics would be of interest?

What is the best way to get people to watch the videos?

**4) Tell me about the sites you like to go to for information about caring for your baby and parenting**

Prompts to consider

What do you use the site for?

Describe how you interact with the site.

Creating content, sharing and/or getting information/advice/support.

What is most helpful about the site?

Tell me about an example of a post that was very helpful to you.

What is unhelpful about the site?

How would you change it?

Tell me an example of a post that was unhelpful to you.

Tell me about your usage

Frequency of use.

Time of day for use.

Regarding trust or distrust about what you read on the site (broadly).

Do you trust what you see on these sites? All of the sites, or some?

Why?

What makes you trust the sites you do trust?

What stands out that makes you trust it?

Why don’t you trust this site (these sites)?

What makes you not trust this site (these sites)?

Could you tell me about a time you trusted information? Could you tell me about a time you distrusted information?

What created with trust?

What creates distrust?

What did you learn from it?

Engagement

Describe how you engage (interact) with the site.

Describe how you engage (interact) with the participants.

Tell me about a time when there was a post that you did not agree with?

What did you do?

How did you feel?

Ask above prompts for each site.

**Tell me about the sites you like(d) to go to for pregnancy information.**

Prompts to consider

What did you use the site for?

Describe how you interacted with the site.

Creating content, sharing and/or getting information/advice/support.

What was most helpful about the site?

Tell me about an example of a post that was very helpful to you.

What was unhelpful about the site?

How would you change it?

Tell me an example of a post that was unhelpful to you.

Tell me about your usage

Frequency of use.

Time of day for use.

Regarding trust or distrust about what you read on the site. Could you tell me about a time you trusted information? Could you tell me about a time you distrusted information?

What created with trust?

What creates distrust?

What did you learn from it?

Engagement

Describe how did you engaged (interacted) with the site.

Describe how you engaged (interacted) with the participants.

Tell me about a time when there was a post that you did not agree with?

What did you do?

How did you feel?

Ask above prompts for each site.

*Before we move on to another topic – do you have any other comments about what we have been discussing?*

*Now I want to switch to discussing about how you access the sites.*

**Tell me about the different ways that you access the sites you use.**

Prompts to consider

What does a typical day of social media use look like for you?

What apps/sites do you usually have open?

Computer access through website, app on the phone, tablet etc.

Why do you choose this/these route for access?

Tell me things that might get in the way of you accessing social media.

Tell me things that make it easy for you to access social media.

4. Summary

Give a brief summary of what has been discussed and ask:

Is this an adequate summary?

5. Final question

Review the reason for the interview and then ask if we have missed anything during the discussion that is important for the topic.

*Thank you for participating in this interview.*

*Before we stop, is there anything else you would like to add?*

*And we wanted to see if you would be willing to be contacted again in the future, for example to check in with you about or findings?*
